# Anti-aggregation Effects of Phenolic Compounds on α-synuclein

**DOI:** 10.3390/molecules25102444

**Published:** 2020-05-24

**Authors:** Kenjiro Ono, Mayumi Tsuji, Tritia R. Yamasaki, Giulio M. Pasinetti

**Affiliations:** 1 Division of Neurology, Department of Internal Medicine, School of Medicine, Showa University, Tokyo 142-8666, Japan; 2 Pharmacological Research Center, Showa University, Tokyo 142-8666, Japan; tsujim@med.showa-u.ac.jp; 3 Department of Neurology, University of Kentucky, Lexington, KY 40536, USA; tyamasaki@uky.edu; 4 Department of Neurology, Icahn School of Medicine at Mount Sinai, New York City, NY 10029, USA; giulio.pasinetti@mssm.edu

**Keywords:** Parkinson’s disease, α-synuclein, phenolic compounds, gut microbiome

## Abstract

The aggregation and deposition of α-synuclein (αS) are major pathologic features of Parkinson’s disease, dementia with Lewy bodies, and other α-synucleinopathies. The propagation of αS pathology in the brain plays a key role in the onset and progression of clinical phenotypes. Thus, there is increasing interest in developing strategies that attenuate αS aggregation and propagation. Based on cumulative evidence that αS oligomers are neurotoxic and critical species in the pathogenesis of α-synucleinopathies, we and other groups reported that phenolic compounds inhibit αS aggregation including oligomerization, thereby ameliorating αS oligomer-induced cellular and synaptic toxicities. Heterogeneity in gut microbiota may influence the efficacy of dietary polyphenol metabolism. Our recent studies on the brain-penetrating polyphenolic acids 3-hydroxybenzoic acid (3-HBA), 3,4-dihydroxybenzoic acid (3,4-diHBA), and 3-hydroxyphenylacetic acid (3-HPPA), which are derived from gut microbiota-based metabolism of dietary polyphenols, demonstrated an in vitro ability to inhibit αS oligomerization and mediate aggregated αS-induced neurotoxicity. Additionally, 3-HPPA, 3,4-diHBA, 3-HBA, and 4-hydroxybenzoic acid significantly attenuated intracellular αS seeding aggregation in a cell-based system. This review focuses on recent research developments regarding neuroprotective properties, especially anti-αS aggregation effects, of phenolic compounds and their metabolites by the gut microbiome, including our findings in the pathogenesis of α-synucleinopathies.

## 1. Introduction

Parkinson’s disease (PD) is the most common type of parkinsonism, a term reflecting a group of neurological disorders that cause PD–like movement problems such as rigidity, slowness, and tremor. More than six million individuals worldwide have PD [1]. The disease is characterized by the death of dopaminergic neurons in the substantia nigra. The pathologic hallmark of PD is the Lewy body (LB), a neuronal inclusion consisting largely of α-synuclein (αS) protein aggregations, which are associated with the death of dopamine-producing cells. The most widely cited model for explaining the neuropathological progression of PD is the Braak hypothesis [2]. LB pathology starts (stages 1 and 2) in the medulla and the olfactory bulb. This early pathology is associated with symptoms that occur before the onset of movement difficulties. These early symptoms include rapid eye movement sleep behavior disorder and decreased smell. In stages 3 and 4, the pathology progresses to the substantia nigra pars compacta and other midbrain and basal forebrain structures. Pathology in these areas is associated with classic motor symptoms, and, typically, PD is diagnosed at this stage. In advanced PD, the pathology progresses to the cerebral cortices and is concomitant with the onset of cognitive impairment and hallucinations [2].

In rare cases, autosomal dominant PD is caused by missense variants (A53T, A30P, and E46K) [3,4,5] and the overexpression of αS [6,7]. Currently, the supplementation of dopamine is the mainstay of PD treatment, since, to date, no means to modify αS aggregation have been identified [1]. Dementia with Lewy bodies (DLB) is a slowly progressive and persistent dementia disorder of the elderly, clinically characterized by fluctuating attention, recurrent visual hallucinations, and parkinsonism [8,9]. As with PD, LBs and Lewy neurites in the brain constitute the main histopathological features of DLB.

Multiple system atrophy (MSA) is a neurodegenerative disease characterized by progressive autonomic failure, parkinsonism, and cerebellar and pyramidal tract symptoms. Glial cytoplasmic inclusions of αS are a defining histologic feature of this disease, and there is no curative treatment [10]. An in vivo study in mice demonstrated that the overexpression of αS in oligodendrocytes results in MSA-like degeneration in the central nervous system [11]. Taken together with biochemical and genetic evidence, the aggregation of αS may play an important role in the development of α-synucleinopathies, including PD, DLB, and MSA.

αS tend to fold and aggregate to form oligomers, protofibrils, and mature fibrils; it has been suggested that these cause neuronal dysfunction in the pathogenesis of α-synucleinopathies [12,13]. Although the internal origin of αS toxicity remains unclear, accumulating evidence suggests that it may be the oligomeric forms of αS, rather than the larger intracellular inclusions (mature fibrils), that are the most bioactive and, possibly, cytotoxic, causing not only neuronal dysfunction but also cell death [14,15,16,17].

**Figure 1:**
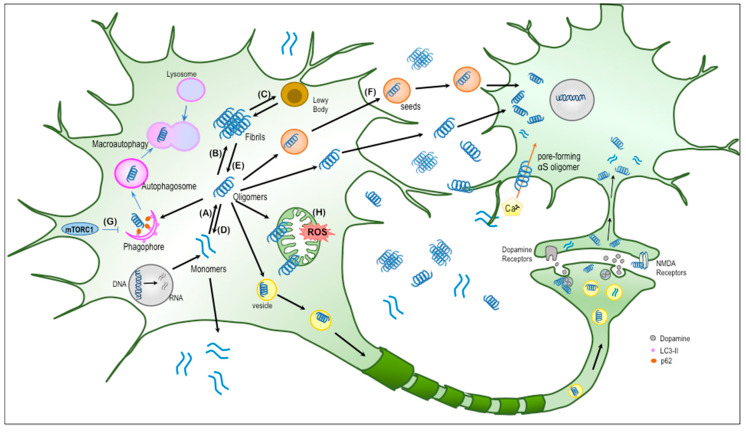
The main effects of natural phenolic compounds modulating αS aggregates. (**A**,**B**) Inhibiting the oligomerization and fibrillization of αS, (**C**) preventing the accumulation of αS fibrils, (**D**,**E**) promoting the degradation of αS fibrils, (**F**) preventing the seeding and transfer of αS from cell to cell, (**G**) downregulating mTORC1 (mammalian target of rapamycin complex 1) signaling and recovering suppressed macroautophagy, and (**H**) reducing ROS (reactive oxygen species) generation.

Dietary intake of polyphenols may be associated with PD risk. A large prospective study conducted over two decades involving almost 130,000 individuals showed that the habitual intake of polyphenols and polyphenol-rich foods such as berry fruits may reduce the risk of developing PD, and the association is more pronounced in men than in women [18]. Previous epidemiological studies have detailed an inverse relationship between green/black tea consumption and the risk of developing PD [19,20,21,22,23]. Moreover, a meta-analysis of tea drinkers and non-drinkers showed that tea drinking offers protective effects against the risk being affected by PD [24]. The protective effects have been attributed to the antioxidant and anti-inflammatory properties of these foodstuffs [25]. Evidence from in vitro and in vivo studies has further indicated that polyphenols such as (–)-epigallocatechin gallate (EGCG) in green tea, curcuminoids in curry, baicalein extracted from the root of *Scutellaria baicalensis*, a traditional Chinese herb, or extracts from grape and blueberry protect against neuronal damage in PD [26,27]. Levites et al. reported that EGCG shows neuroprotective properties against 1-methyl-4-phenyl-1,2,3,6-tetrahydropyridine (MPTP)-induced parkinsonism in animal models because of the iron-chelating and free radical scavenging activities of the cathecol group [28,29]. Polyphenols also have protective effects against αS toxicity [30,31]. In experiments involving cell models of PD, curcumin (Cur) was shown to reduce αS-induced cytotoxicity by decreasing intracellular reactive oxygen species (ROS), mitochondrial depolarization, cytochrome c release, and caspase-9 and caspase-3 activation [30], or by downregulating mTOR (mammalian target of rapamycin)/p70S6K signaling and recovering macroautophagy [31] (Figure 1).

Previously, we demonstrated that phenolic compounds such as myricetin (Myr), Cur, rosmarinic acid (RA), nordihydroguaiaretic acid (NDGA), and ferulic acid (FA) inhibit the formation of αS fibrils and destabilize preformed fibrils [27]. Similarly, it was reported that baicalein [32] and EGCG also inhibit αS fibrillization and destabilize preformed fibrils [33,34]. Later, we revealed that Myr and RA hinder αS oligomerization and secondary structure conversion, thus ameliorating αS synaptic toxicity. Similarly, it has been reported that EGCG exerts protective effects against αS oligomer-induced membrane disruption and cytotoxicity by facilitating fibril formation and eliminating toxic αS oligomers [35]. These results suggest that phenolic compounds prevent the occurrence of αS aggregation, thereby reducing the neurotoxicity of αS oligomers.

A number of studies have reported the presence of gut microbiota dysbiosis in patients with PD, suggesting that this is a risk factor for developing the illness [36,37,38]. Moreover, recent preclinical observations have supported a causal relationship between gut microbiota dysbiosis and PD pathophysiology [39,40,41]. Notably, phenolic compounds modulate the gut–brain axis, which transforms them into neuroprotective compounds through gut microbiome metabolism [42]. Recently, we observed that interpersonal heterogeneity in gut microbiota may lead to interpersonal variabilities in the efficacy with which dietary polyphenols are metabolized into select biologically available phenolic metabolites [43]. We demonstrated that the brain-accumulating phenolic metabolites identified in cecum specimens of gnotobiotic mice, namely, 3-(3′-hydroxyphenyl)propionic acid (3-HPPA), 3,4-dihydroxybenzoic acid (3,4-diHBA), 3-hydroxybenzoic acid (3-HBA), and 4-HBA, inhibit αS oligomerization in vitro [43]. These phenolic acids also improved behavioral disturbances in a Drosophila model of α-synucleinopathy [43]. Very recently, we determined not only that 3-HPPA, 3,4-diHBA, 3-HBA, and 4-HBA significantly attenuate intracellular αS seeding aggregation in a cell-based system but also, using insoluble αS seeds extracted from post-mortem MSA or PD brain specimens, 3-HPPA effectively attenuates the MSA-induced intracellular aggregation of αS [44].

This review focuses particularly on recent research developments regarding the neuroprotective properties, especially the anti-αS aggregation effects, of phenolic compounds and the ability of phenols and their metabolites to cross the blood–brain barrier. Our findings on the pathogenesis of α-synucleinopathies are also included.

## 2. α-Synuclein Aggregation

α-Synuclein tends to self-aggregate or cause the aggregation of other proteins. In vitro studies have shown both full-length wild-type (WT) and mutant αS molecules to be capable of self-aggregation into mature fibrils in a process that is dependent on time, temperature, pH, and concentration [45,46]. In these studies, the 10 nm-wide fibrils were noticeably twisted and bore a close resemblance to the fibrils isolated from both the LBs of patients with PD and DLB and the filamentous inclusions characteristic of MSA [47,48,49]. Until the early 2000s, αS fibrillization was thought to be a critical step in the pathogenesis of α-synucleinopathies [2,50]. Subsequently, a change in secondary structure from an unfolded random coil to an antiparallel β-sheet structure has been shown to accompany the fibrillization of αS [51,52].

**Figure 2:**
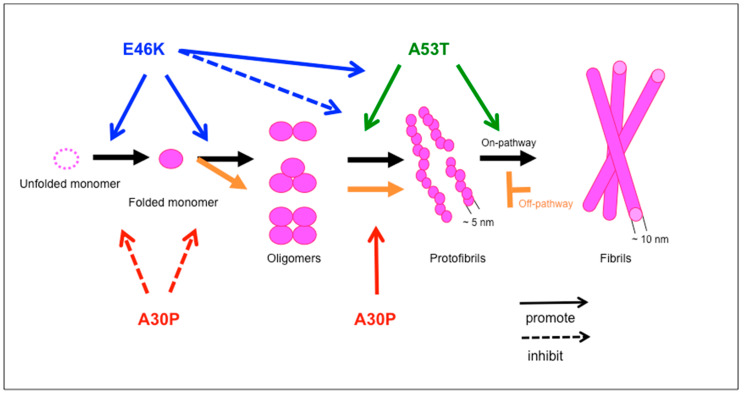
αS mutants, including E46K, A53T, and A30P influence some stages of αS aggregation.

Comparable with the fibrillization of amyloid β-protein (Aβ) in vitro [53,54], the time course of αS fibrillization fulfills all the criteria of the nucleation-dependent model, characterized by an initial lag phase that reflects nucleation (seed formation) and a subsequent growth phase that culminates in a steady state [55]. A conformational change in αS may result in the formation of misfolded intermediates such as β-sheet oligomers or protofibrils (Figure 2) [55]. These αS aggregates can act as the “seeds” in a nucleation-dependent model of αS fibrillization. A recent study revealed that αS fibrils grow by monomer addition rather than oligomer addition and are subject to higher-order assembly processes that decrease their capacity to grow [56]. It was also found that, at neutral pH, the growth of αS aggregates and higher-order assembly of fibrils occur at much greater rates than either primary nucleation or secondary processes. However, at mildly acidic pH values, secondary nucleation is strongly accelerated, changing the mechanistic characteristics of αS aggregation. Thus, at mildly acidic pH values—such as those, for example, that are present in some intracellular locations, including endosomes and lysosomes—the multiplication of aggregates occurs much more quickly than at normal physiological pH values, largely as a consequence of much more rapid secondary nucleation [56].

As with WT αS, the fibrillization of mutated αS follows a nucleation-dependent model dose-dependently [52,57,58,59]. αS mutants, including A53T, A30P, and E46K, have been reported to influence some stages of αS aggregation in vitro (Figure 2) [46,49,57,58,60,61,62]. For example, first, the A53T mutation increases the propensity for αS fibrillization, as well as the formation of protofibrils [46,49,52]. Second, the A30P mutation promotes the formation of protofibrils but not αS fibrils [52,60]. Some of these protofibrils, which have circular morphology, form pores by binding to the endoplasmic reticulum membrane [13]. Third, the E46K mutation has also been reported to increase the propensity for fibrillization in vitro [61] and to reduce the propensity for protofibrillization by reducing the permeability of lipid vesicles [57]. In our previous in vitro study, E46K αS accelerated the kinetics of the secondary structure changes and oligomerization, whereas A30P αS decelerated these early changes compared with WT αS [58]. Similarly, the oligomers of E46K αS functioned as fibril seeds significantly more efficiently than those of the WT αS, whereas the oligomers of A30P αS were less efficient. These results demonstrated that αS mutations have opposite effects at the earliest stage of αS assembly [58]. Recently, a detailed analysis using X-ray diffraction pattern recording, circular dichroism, Fourier-transform infrared spectroscopy, electron microscopy, and atomic force microscopy, demonstrated that the subgrouping of different mutational variants from a kinetic perspective correlates with the subgrouping of the structural and morphological features of the resulting fibrils. A53T and A30P variants show similar kinetic constants, structure, and morphology, when compared with the WT protein, whereas the E46K, H50Q, and G51D variants show large differences in secondary structure, morphology, and microscopic steps in the aggregation mechanism, relative to the WT protein. These results indicate that the mechanism underlying the amyloid formation, morphology, and structure of fibrillar aggregates is generally correlated in all variants of αS. Thus, a single point mutation can significantly alter the distribution of fibrillar polymorphs of αS, suggesting that differences in the clinical phenotypes of familial PD could be associated with changes to the mechanism of formation and the particular structural characteristics of the aggregates [59].

## 3. Toxicity of αS Oligomers

In vitro and in vivo studies support the oligomer hypothesis [12,17,52]. In vitro studies have shown that annular protofibrils alter membrane permeability, resulting in an increased influx of calcium from the extracellular to the intracellular space, leading to cell death [63,64]. Similarly, annular low-n oligomers (mainly octamers) have been reported to form pore-like structures that fully perforate the lipid layer of membranes with resultant calcium efflux; A53T αS shows a greater tendency to establish this type of permeability than WT αS [65]. The study also demonstrated that transgenic mice highly expressing αS E57K show abundant oligomeric, but not fibrillar, αS; exacerbated synaptic and dendritic loss; diminished levels of synapsin 1 and synaptic vesicles; and behavioral deficits. This suggests that accumulating αS oligomers may mediate early synaptic pathology in α-synucleinopathies by disrupting synaptic vesicles [66]. Similarly, it has been shown, using the brains of transgenic mice overexpressing αS and in a study of humans with LB dementia, that αS oligomers are associated with the loss of several critical presynaptic proteins, which results in dysfunctional synapses and eventual neurodegeneration [67]. Moreover, it has been reported that oligomeric αS inhibits long-term potentiation through an increase in intracellular calcium levels, induces calcineurin activity, reduces the cyclic AMP (adenosine monophosphate) response element-binding protein transcriptional activity in ex vivo rat brain slices, and evokes memory impairments in mice [14]. Recently, Knowles’s group used single-molecule techniques to measure the equilibrium populations of oligomers formed in vitro by mixtures of WT αS and mutational αS variants. They discovered that co-oligomer formation is generally more favorable than self-oligomer formation at equilibrium [64]. Moreover, self-oligomers disrupt lipid membranes more potently than do co-oligomers. However, this adverse effect can dominate when co-oligomers are present at high steady state concentrations [64]. In 2019, Ghio et al. used single-channel electrophysiology to observe that the presence of cardiolipin, the signature phospholipid in mitochondrial membranes, enhances αS-lipid interaction and the membrane pore-forming activity of αS oligomers [68] (Figure 1). Furthermore, they identified that the preincubation of isolated mitochondria with cardiolipin-specific dye protects against αS oligomer-induced mitochondrial swelling and the release of cytochrome c. This finding indicates that αS oligomers directly porate local lipid environments rich in cardiolipin, such as outer mitochondrial contact sites or the inner mitochondrial membrane, to induce mitochondrial dysfunction [68]. Similarly, Ahyayauch et al. revealed that the interaction between the Aβ monomer and membrane becomes stronger in the presence of negatively charged cardiolipin using both biophysical and computational techniques [69]. In 2020, McLean’s group demonstrated that mitochondrial sirtuin 3 (SIRT3) downregulation is accompanied by decreased phosphorylation of AMP-activated protein kinase (AMPK) and cAMP-response element-binding protein, as well as increased phosphorylation of dynamin-related protein 1, which is indicative of impaired mitochondrial dynamics in cells expressing oligomeric αS within the cytosolic and mitochondrial-enriched fractions. Interestingly, treatment with the AMPK agonist 5-aminoimidazole-4-carboxamide-1-β-d-ribofuranoside restores SIRT3 expression, improves mitochondrial function, and decreases αS oligomer formation SIRT3-dependently. These findings suggest that pharmacologically increasing SIRT3 levels can counteract αS oligomer-induced mitochondrial dysfunction by reducing the number of αS oligomers and normalizing mitochondrial bioenergetics [70]. Recently, Spillantini’s group interestingly showed that treatment with the oligomer modulator anle138b restores striatal dopamine release and prevents dopaminergic cell death and gait impairment in a new transgenic mouse line, MI2, which exhibits progressive αS aggregation in dopaminergic neurons of the substantia nigra pars compacta and their striatal terminals. These effects were associated with a reduction in the inner density of large αS aggregates and an increase in dispersed small αS species, as revealed by dSTORM. These data provide a new mechanistic insight into the effect of anle138b’s function in vivo, which supports the targeting of αS oligomerization as a promising therapeutic approach for α-synucleinopathies [71].

## 4. Propagation of αS Aggregates Including Oligomers

α-Synuclein pathology exhibits an ascending distribution of the LB pathology in LB diseases, as described by Braak, beginning in the lower brainstem and anterior olfactory nucleus, progressing to midbrain and forebrain nuclei, and finally involving the cerebral cortex [2,72]. Cell-to-cell transfer and seeding are other mechanisms by which αS pathology is suggested to propagate through the brain [73]. Notably, evidence includes the observation that healthy dopaminergic neurons transplanted into PD brains can eventually form LBs [74,75,76]. This prion-like transmission of pathological seeds from host cells to graft cells has been replicated in in vivo models [77,78].

Experimental evidence has repeatedly shown αS seeding to spread from the striatum to dopaminergic neurons in the substantia nigra pars compacta, which innervates the striatum [79,80,81], causing the death of these neurons [79,81] and resulting in a substantial reduction in striatal dopamine levels and impaired motor coordination [79].

Another way in which αS resembles Aβ and tau is the existence of multiple conformational strains with distinct biochemical properties and varying seeding effects and toxicity. Tau strains are also essential for the seeding and propagation of tau pathology, and they target different brain regions [82]. Guo et al. demonstrated that two different strains of preformed αS fibrils can be generated, one of which is highly effective at cross-seeding tau aggregation both in vitro in cultured cells and in vivo in PS19 mice [83]. We previously examined whether sonicated fibrils or oligomers of αS, Aβ_1–40_, and Aβ_1–42_ affected their aggregation pathways in vitro. These fibrils and oligomers acted as seeds and affected the aggregation pathways within and among species, suggesting the possibility of molecular interaction in propagation between the pathologies of α-synucleinopathies, Alzheimer’s disease, and tauopathies [84]. Recently, Knowles’s group determined that co-oligomer formation of WT αS with Aβ_1–40_, Aβ_1–42_, and tau is generally more favorable than self-oligomer formation at equilibrium in vitro, suggesting that co-oligomer formation may also be important in the mixed pathologies of the above-described neurodegenerative diseases [64].

α-Synuclein pathology becomes more severe with time and spreads to brain regions outside the injection site after intracerebral seeding, as with seeded Aβ or tau pathology [85]. There is strong evidence that the pattern of spread is dictated by connectivity [79,80] and is consistent with a mechanism of anterograde and retrograde cell-to-cell transmission [86]. Evidence of trans-synaptic αS propagation mediated by αS seeds crossing the synapse between first- and second-order neurons has also been observed, with pathology appearing over time in regions indirectly connected to the injection site [79,86,87]. Recent evidence suggests that toxic αS oligomers may be released from neurons via unusual secretory mechanisms, such as exocytosis, in the pathogenesis of α-synucleinopathies [88,89]. Interestingly, these extracellular αS oligomers can then transfer between neurons or from neuron to glial cells [90], where they can nucleate further intracellular aggregation, leading to neuroinflammation and exacerbation of the neurodegenerative process [91,92]. The mechanisms through which extracellular αS oligomers transfer to other cells include endocytosis [91,92], direct penetration [15], trans-synaptic dissemination [93], and membrane receptor-mediated access [90]. Once inside the acceptor cell, αS oligomers can act as trigger points for further intracellular aggregation, or the protein can be targeted for degradation. As discussed earlier, evidence from in vitro biophysical studies has consistently shown that the fibrillization of αS follows a nucleated polymerization mechanism [51,55,58]. This mechanism is characterized by a nucleation phase that initially involves the formation of oligomers (acting as the seeds), followed by cooperative oligomer growth and fibrillization by monomer addition [84]. This process can be thought of as the mechanism that underlies the spread of αS pathology in the brain (Figure 1). The process has been observed in a cell-based assay, in which the induction of recombinant αS fibrils resulted in seeding, the recruitment of endogenous αS, and the formation of LB-like inclusions [94]. A recent in vivo study has shown that the inoculation of αS transgenic mice with homogenates containing αS protofibrils and fibrils results in a considerable enhancement of αS pathology and propagation [80]. To directly visualize and characterize αS oligomerization and spreading in vivo, Danzer’s group recently generated two independent conditional transgenic mouse models, based on αS protein complementation assays, using neuron-specifically expressed split Gaussia luciferase or split Venus yellow fluorescent protein (YFP) [95]. Using these mouse models, they demonstrated the age-dependent accumulation of a specific subtype of αS oligomer in vivo. They also provided in vivo evidence that, although αS is found throughout the neurons, αS oligomerization occurs at the presynapse, and de novo generated αS oligomers are transferred via a trans-synaptic cell-to-cell pathway in vivo [95] (Figure 1). These observations have led to the challenging hypothesis that extracellular αS seeds may participate in the prion-like propagation of neurodegeneration in α-synucleinopathies [12]. Further experimental evidence is needed to confirm this hypothesis.

## 5. Antioxidant Properties of Phenolic Compounds 

Most polyphenols are salubrious because of their potent antioxidant nature. These agents can neutralize free radicals via hydrogen atom abstraction [96]. Polyphenol transformation products with free radicals produced by radical quenching can further react with secondary free radicals, leading to the formation of a stable quinone structure [96]. Generally, their radical scavenging ability depends on the molecular structure and substitution pattern of the hydroxyl group. Besides this ability, polyphenols can also bind to metal ions, which may further enhance their antioxidant activity [96]. EGCG, an antioxidant and metal-chelating polyphenol in green tea, was shown to regulate the iron-export protein ferroportin in the substantia nigra, reduce oxidative stress (measured as protein carbonyls in serum), and finally exert a neurorescue effect against MPTP-induced motor deficits in mice [28]. Similarly, Ryu et al. screened HEK293 cells that stably express the microtubule-associated protein light chain 3 (LC3) protein, a marker of autophagy, for autophagy activity in 100 single plant compounds, and identified amurensin G, a compound isolated from the wild grape (*Vitis amurensis*) [97]. Treatment with amurensin G induced the punctate cytoplasmic expression of green fluorescent protein (GFP)-LC3 and increased the expression of endogenous LC3-II (Figure 1). The incubation of human dopaminergic SH-SY5Y cells with amurensin G attenuated rotenone-induced cellular toxicities by reducing the level of ubiquitinated proteins and αS. Moreover, amurensin G inhibited rotenone-induced apoptosis and interfered with the rotenone-induced G2/M cell cycle arrest. With the added finding that the knockdown of beclin1, a regulator of autophagy, abolished the effect of amurensin G, this compound was suggested to attenuate neurotoxicity through the induction of autophagy in a cellular model of PD [97]. A botanical extract prepared from grape (*Vitis vinifera*) and *Polygonum cuspidatum*, which contains polyphenols (including flavans, anthocyanins, emodin, and resveratrol), exhibited dose-dependent scavenging effects on ROS (Figure 1). The extract inhibited increases in ROS and protein carbonyl in isolated rat liver mitochondria following exposure to 2,2′-azobis (2-amidino propane) dihydrochloride (AAPH), a potent lipid oxidant generator. The antioxidant effects of this extract were further demonstrated by protecting the enzyme activities of the mitochondrial respiratory electron transport chain (complexes I and II) and pyruvate dehydrogenase in isolated liver mitochondria with AAPH insult. The pretreatment of human neuroblastoma cells (SKN-MC) with extract induced oxidation to maintain cell viability while inhibiting excessive ROS generation. The extract was also fed to transgenic human αS-expressing Drosophila models that produce adult-onset loss of dopaminergic neurons, filamentous intraneuronal inclusions containing αS, and locomotor dysfunction. Male transgenic flies fed with the extract showed a significant improvement in climbing ability, compared with controls, using a geotaxis assay. Furthermore, female transgenic flies showed a significant extension in average lifespan. Similarly, the consumption of grape skin extract containing resveratrol was reported to result in the rescue of mitochondrial morphological defects, improvement of indirect flight muscle function and healthspan, and prolonged lifespan in a *Drosophila melanogaster* model of PD associated with phosphatase tensin homolog-induced kinase 1 loss of function [98]. These results suggest that botanical extracts containing a variety of polyphenols are potent free radical scavengers and mitochondrial protectors that protect against neurodegeneration and potentially extend lifespan in a PD model [99].

## 6. Inhibition of αS Fibrillization by Phenolic Compounds

Over the last two decades, various phenolic compounds have been tested extensively for their ability to inhibit αS aggregation. It has been clearly shown that certain polyphenols can dramatically inhibit cell death induced by αS aggregates [100,101,102]. To assist in developing a disease-modifying approach for α-synucleinopathies focused on the aggregation of αS, our group and others have reported that various phenolic compounds such as wine-related polyphenol [27,32], NDGA [27], rifampicin [103], and Cur [27] inhibit αS fibrillization and destabilize preformed fibrils. In our first study of αS, compounds with anti-fibrillogenic and fibril-destabilizing activity were ranked in the following order: tannic acid = NDGA = Cur = RA = Myr > kaempferol = FA > (+)-catechin = (–)-epicatechin > rifampicin = tetracycline [27]. NDGA, Cur, and RA are smaller than rifampicin and have two 3,4-dihydroxyphenyl rings (NDGA and RA) or 4-hydroxy-3-methoxyphenyl rings (Cur) symmetrically bound by a short hydrocarbon chain. Similarly, FA contains one 4-hydroxy-3-methoxyphenyl ring and has been identified as a degradation product of Cur [104]. This compact structure may be quite suited specifically to binding free αS monomers and, subsequently, inhibiting the polymerization of peptides into fibrils. Alternatively, this structure might be suited to binding preformed fibrils of αS and, subsequently, destabilizing the β-sheet rich conformation of these molecules in fibrils [27]. We speculated that the difference in the three-dimensional structure and the numbers of hydroxyl groups of these phenolic compounds would greatly affect binding to αS monomers and/or αS fibrils in anti-aggregation and fibril-destabilizing activity (Ono et al. 2006). Another systematic study showed that polyphenols such as Cur, baicalein, EGCG, and resveratrol in combination with β-cyclodextrin not only synergistically inhibited αS aggregation but were also effective in disaggregating preformed fibrils at substoichiometric concentrations of the individual components, resulting in the reduced toxicity of prefibrillar αS aggregates on mouse neuroblastoma cell lines (N2a cells) [105]. In recent work, EGCG was shown to form a Cu(II)/EGCG complex, leading to the inhibition of the Cu(II)-induced conformation transition of αS from random coil to β-sheet, which is a dominant structure in αS fibrils and aggregates [106]. Moreover, the mixture of Cu(II) and EGCG in a molar ratio from 0.5 to 2 efficiently inhibited this process. Furthermore, EGCG inhibited the overexpression and fibrillization of αS in αS-transduced PC12 cells and reduced Cu(II)-induced ROS, protecting the cells against Cu(II)-mediated toxicity [106]. Cur was found to be the most efficient of the polyphenols investigated, followed by baicalein, EGCG, and resveratrol, with the latter two compounds exhibiting very similar effects. The authors suggested that the efficiency of Cur results from a balanced composition of the phenolic OH groups, benzene rings, and flexibility. The latter ensures the proper positioning of the functional groups to maximize underlying interactions with both the monomer of αS and its aggregates [105].

Fink’s group (2004) previously reported that, at low micromolar concentrations, baicalein not only inhibits αS fibrillization but also disaggregates preformed αS fibrils [32]. They suggested that the product of the inhibition reaction is predominantly a soluble oligomer of αS, in which the protein molecules have been covalently modified by the binding of baicalein quinone to form a Schiff base with a lysine side chain in αS [32]. Similarly, Li et al. (2004) showed that rifampicin, having a phenolic structure, also inhibits αS fibril formation at substoichiometric low micromolar concentrations and disaggregates preformed αS fibrils by covalent binding to stabilize αS monomers and oligomers on the off-pathway [103]. Similarly, Ehrnhoefer et al. (2008) reported that EGCG efficiently inhibits αS fibrillization by stabilizing non-toxic oligomers on the off-pathway differently from those on the on-pathway, through the direct binding of the unfolded monomer [33,34]. More recently, EGCG-mediated protection against αS oligomers has been reported to reduce membrane disruption and subsequent cellular degeneration by facilitating the conversion of on-pathway toxic αS oligomers into fibrils and thus accelerating the removal of toxic oligomers [35]. Furthermore, Xu et al. demonstrated that EGCG inhibits αS aggregation concentration-dependently using three methods: αS fibril formation inhibition by thioflavin T binding in vitro, inhibition of αS fluorophore αS-HiLyte488 binding to plated αS in a microplate assay, and inhibition of αS -HiLyte488 probe binding to aggregated αS in a post-mortem PD tissue-based assay [107]. The αS amino acid sites, which potentially interact with EGCG, were detected on peptide membranes, implying that EGCG binds to αS through instable hydrophobic interactions [107]. El-Agnaf’s group showed that gallic acid (GA, 3,4,5-trihydroxybenzoic acid), a benzoic acid derivative that belongs to a group of phenolic compounds known as phenolic acids, not only inhibits αS fibrillization and toxicity but also disaggregates preformed αS fibrils. Interestingly, GA was also found to bind to soluble, non-toxic oligomers with no β-sheet content and to stabilize their structure [101]. The structure activity relationship data obtained from 14 structurally similar benzoic acid derivatives demonstrated that the inhibition of αS fibrillization by GA is related to the number of hydroxyl moieties and their position on the phenyl ring, which concurs with our data [27,101]. Macedo et al. evaluated the effects of a polyphenol-enriched fraction (PEF) from the leaves of *Corema album* on αS toxicity and aggregation in vitro and using αS-expressing cellular models. The PEF promoted the formation of non-toxic αS oligomers in vitro and inhibited αS toxicity and fibrillization in cells by stimulating autophagy and reducing oxidative stressors such as H_2_O_2_ [102].

In animal models of PD, several studies have clearly demonstrated that various phenolic compounds possess neuroprotective effects, but no study has investigated the interaction between polyphenols and αS aggregation in vivo [108]. Using transgenic mice with overexpressed human GFP-tagged WT αS, a Cur-containing diet was shown to improve motor behavior with an increase in phosphorylated αS, but no effect on αS aggregation was seen in vivo [109]. 

## 7. Inhibition of αS Oligomerization by Phenolic Compounds

In relation to the oligomer hypothesis for the pathogenesis of α-synucleinopathies, we and other groups showed that phenolic compounds inhibit αS oligomerization and reduce cytotoxicity in vitro and in vivo. Cur, the main component of turmeric spice, was reported to both inhibit and reverse the formation of high-order toxic αS oligomers by directly binding to αS in vitro and in cell culture models [110]. Subsequently, Cur was demonstrated to intra- and extra-cellularly alleviate αS oligomer-induced toxicity by reducing the levels of ROS and inducing apoptosis in SH-SY5Y cells [111]. Using a series of biophysical techniques, Maji’s group demonstrated that Cur reduces toxicity by binding to αS oligomers and fibrils on-pathway, and that reducing the solvent exposed their hydrophobic surface [112]. Their fluorescence and two-dimensional nuclear magnetic resonance (NMR) data indicated that Cur does not bind to monomeric αS but instead binds specifically to oligomeric intermediates. The degree of Cur binding correlates with the extent of αS oligomerization suggests that an ordered protein structure is required for effective Cur binding. The acceleration of αS aggregation by less toxic off-pathway oligomers altered by Cur may decrease the population of toxic αS oligomers on-pathway [112]. Later, using biochemical, biophysical, and cell-based assays, it was discovered that Cur pyrazole and its derivative N-(3-nitrophenylpyrazole) Cur exhibit remarkable potency in not only inhibiting fibrillization and disrupting preformed αS fibrils but also preventing the formation of A11 reactive αS oligomers that impart toxic effects by promoting stabilization of off-pathway oligomers. The compounds also decreased the neurotoxicity associated with fast aggregating A53T αS in SH-SY5Y cells [113].

Recently, resveratrol was found to inhibit αS aggregation in vitro and reduce αS oligomer-induced cytotoxicity in SH-SY5Y cells, while alleviating motor and cognitive deficits in the A53T αS mouse model of PD, by lowering the levels of total αS and oligomers; reducing neuroinflammatory cytokines such as tumor necrosis factor-α, interleukin (IL)-1β, and IL-6; and limiting oxidative stress factors such as ROS and malondialdehyde in vivo. 

Using methods such as the photo-induced cross-linking of unmodified proteins (PICUP), circular dichroism spectroscopy, electron microscopy, and atomic force microscopy, we previously reported that, dose-dependently, Myr and RA inhibit a low-n order oligomerization and secondary structure transition of αS including random coil → β-sheet [114]. Similarly, the polymeric wine-related polyphenol, tannic acid, was reported to display inhibitory effects on αS oligomerization and disaggregate preformed αS oligomers in vitro [115]. Our detailed NMR-based investigation revealed that Myr directly binds to the N-terminal region of αS, whereas direct binding of RA to monomeric αS was not detected. More recently, quercetin was also shown to bind monomeric αS covalently, with the increased hydrophilicity of the covalently modified αS resulting in the inhibition of further aggregation [116]. Moreover, by recording long-term potentiation in mouse hippocampal slices, our electrophysiological assays demonstrated that these phenolic compounds reduce the synaptic toxicities of αS oligomers [114]. We previously reported similar inhibitory effects of Myr and RA on Aβ oligomerization, resulting in the reduction of Aβ oligomer-induced cellular toxicity and synaptic dysfunction in vitro and in vivo [117,118]. Our NMR analysis of Aβ also showed a similar result in that Myr promoted significant NMR chemical shift changes of monomeric Aβ. These results suggest that phenolic acids may play key roles in blocking the toxicity and early assembly processes associated with both αS and Aβ through different binding strategies [114,118].

Our group demonstrated that rifampicin, with its phenolic structure, inhibits the low-n order oligomerization of Aβ, tau, and αS with the reduction of synapse loss and microglial activation in vitro and in vivo. The intake of rifampicin improved the memory of the mice to a level similar to that in non-transgenic littermates in the Morris water maze. Rifampicin also inhibited cytochrome c release from mitochondria and caspase-3 activation in the hippocampus. Moreover, rifampicin decreased the level of p62/sequestosome-1 in the brain without affecting the increased levels of LC3 conversion, suggesting the restoration of autophagy-lysosomal function (Figure 1). These studies collectively indicate that rifampicin has a broad spectrum of effects and can be used in various pathologies related to oligomers, that is, Alzheimer’s disease, tauopathy, and α-synucleinopathies [119]. Conversely, recent randomized, double-blind, placebo-controlled clinical trial studies have shown that rifampicin and EGCG fail to prevent disease progression in patients with progressive Alzheimer’s disease [120] or with possible or probable MSA [121,122]. Future studies should evaluate rifampicin and other phenolic compounds with anti-oligomeric effects for the early presymptomatic stage (the oligomer phase) of neurodegenerative diseases including α-synucleinopathies.

## 8. Gut Microbiome-Modified Polyphenolic Compounds Inhibit αS Seeding and Spreading in α-Synucleinopathies 

The intestinal microbiota actively converts dietary polyphenols into phenolic acids, some of which are bioavailable in vivo and may promote resilience to select neurological disorders by interfering with key pathologic mechanisms. Since every person harbors a variety of gut bacteria, we previously investigated the influence of the gut microbiota’s interpersonal heterogeneity on the production and bioavailability of polyphenol metabolites that may interfere with αS misfolding. In the study, we generated two experimental groups of humanized gnotobiotic mice with compositionally diverse gut bacteria and orally treated the mice with a polyphenol-rich preparation. The two gnotobiotic mouse groups exhibited distinct differences in the generation and bioavailability of phenol metabolites that show bioactivity in disrupting αS aggregation or reducing inflammation.

We found 15 polyphenol-derived microbial phenolic acid metabolites, including caffeic acid, FA, GA, and vanillic acid at μM to sub-μM concentrations in the cecal compartment across the two gnotobiotic mouse groups. Between the two mouse groups, the breakdown of these metabolites, detected not only in the cecum, colon, and plasma but also in the brain, was different, suggesting that interpersonal heterogeneity in human gut microbiota can drive significant differences in the bioavailability of polyphenol-derived microbial phenolic metabolites in gnotobiotic mice. Three of the 15 biologically available polyphenol-derived metabolites identified in the cecum specimens of gnotobiotic mice, namely, 3,4-diHBA, 3-HBA, and 3-HPPA, accumulate in the brain, although 12 phenolic acid metabolites were detected in plasma specimens from the mice [43]. As with our previous report that 3-HBA and 3-HPPA are effective in preventing the misfolding and assembly of Aβ peptides into neurotoxic aggregates such as Aβ oligomers [123], we showed that 3-HBA, 3,4-diHBA, and 3-HPPA inhibit αS aggregation, including the formation of low-order oligomers such as dimers and trimers, using combination assays with thioflavin dye, electron microscopy, and PICUP. Using the A53T mutant αS Drosophila model of PD, we further investigated the effects of 3-HBA, 3,4-diHBA, and 3-HPPA on modulating PD pathologic phenotypes, in vivo, by monitoring locomotive functions using a negative geotaxis behavior assay (climbing assay) in adult flies [124]. We demonstrated that, in comparison with vehicle-treated mutant flies showing locomotive dysfunction, treatment with 3-HBA, 3,4-diHBA, and 3-HPPA significantly improved the climbing performance of mutant αS-expressing flies [43]. Thus, not only do 3-HBA, 3,4-diHBA, and 3-HPPA reduce the assembly of αS into neurotoxic aggregates in vitro, but these compounds also reduce mutant αS-mediated neurotoxicity in vivo [43]. Very recently, we investigated whether or not 3-HBA, 4-HBA, 3,4-diHBA, or 3-HPPA interfere with αS spreading, in a cell-based system. Using HEK293 cells overexpressing αS-A53T-cyan FP/YFP, we assessed αS seeding activity using Fluorescence Resonance Energy Transfer to detect and quantify αS aggregation. Consequently, we demonstrated that 3-HPPA, 3,4-diHBA, 3-HBA, and 4-HBA significantly attenuate cell-to-cell transfer and intracellular αS seeding aggregation [44] (Figure 1). To determine whether our compounds could inhibit brain-derived seeding activity, we used insoluble αS aggregates extracted from post-mortem MSA or PD brain specimens. We found that 3-HPPA effectively attenuates the MSA-induced aggregation of monomers into high molecular weight aggregates capable of inducing the intracellular aggregation of αS [44]. The outcomes of our studies suggest that the interactions between the gut microbiome and certain phenolic compounds may be effective therapies for modulating pathologic αS aggregation and propagation.

## 9. Conclusions and Future Perspectives

αS aggregation is one of the leading causes of neuronal dysfunction and death in α-synucleinopathies. Current therapies for α-synucleinopathies are limited to symptomatic therapies. The modulation of αS aggregation is emerging as a novel therapeutic target for the treatment of PD. There are two major aspects of αS aggregation that might be targeted therapeutically: first, the protein is prone to aggregate; therefore, anti-aggregative compounds or those that can break pre-existing aggregates may be helpful. Second, there are a number of molecular events, such as aggregation propagation or accumulation of aggregates, that may be targeted therapeutically. There is growing evidence that intermediate aggregates, the soluble oligomers of αS, are proximate neurotoxins. Dietary polyphenols and bioactive metabolites produced by intestinal microbiota are effective not only in the misfolding, oligomerization, and propagation of αS in vitro and in cellular models but also in modulating the development and progression of motor dysfunction in an in vivo model of α-synucleinopathy. If phenolic compounds that target the formation and propagation of toxic αS oligomers reach the clinical stage of investigation in the near future, they have the potential to delay the progression of PD and other α-synucleinopathies. Further clarification of the anti-aggregation effects on αS in the human brain will assist in the development of more effective and safer therapeutics, as well as novel diagnostic assays for α-synucleinopathies.

## Abbreviations

AAPH	2,2′-azobis (2-amidino propane) dihydrochloride
Aβ	amyloid β-protein
AMP	adenosine monophosphate
AMPK	AMP-activated protein kinase
αS	α-synuclein
Cur	curcumin
3,4-diHBA	3,4-dihydroxybenzoic acid
DLB	dementia with Lewy bodies
EGCG	(–)-epigallocatechin gallate
FA	ferulic acid
GA	gallic acid
GFP	green fluorescent protein
3-HBA	3-hydroxybenzoic acid
4-HBA	4-hydroxybenzoic acid
HEK	human embryonic kidney
3-HPPA	3-hydroxyphenylacetic acid
IL	interleukin
LB	Lewy body
LC3	microtubule-associated protein light chain 3
MPTP	1-methyl-4-phenyl-1,2,3,6-tetrahydropyridine
MSA	multiple system atrophy
mTOR	mammalian target of rapamycin
Myr	myricetin
NDGA	nordihydroguaiaretic acid
NMR	nuclear magnetic resonance
PD	Parkinson’s disease
PEF	polyphenol-enriched fraction
PICUP	photo-induced cross-linking of unmodified proteins
RA	rosmarinic acid
ROS	reactive oxygen species
SIRT	sirtuin 3
WT	wild-type
YFP	yellow fluorescent protein
